# A 2:1 co-crystal of 2-methyl­benzoic acid and *N*,*N*′-bis­(pyridin-4-ylmeth­yl)ethanedi­amide: crystal structure and Hirshfeld surface analysis

**DOI:** 10.1107/S2056989016002735

**Published:** 2016-02-20

**Authors:** Sabrina Syed, Mukesh M. Jotani, Siti Nadiah Abdul Halim, Edward R. T. Tiekink

**Affiliations:** a Department of Chemistry, University of Malaya, 50603 Kuala Lumpur, Malaysia; b Department of Physics, Bhavan’s Sheth R. A. College of Science, Ahmedabad, Gujarat 380 001, India; c Centre for Crystalline Materials, Faculty of Science and Technology, Sunway University, 47500 Bandar Sunway, Selangor Darul Ehsan, Malaysia

**Keywords:** crystal structure, co-crystal, hydrogen bonding, carb­oxy­lic acid, di­amide, Hirshfeld surface analysis

## Abstract

The asymmetric unit of the title 2:1 co-crystal, 2C_8_H_8_O_2_·C_14_H_14_N_4_O_2_, comprises an acid mol­ecule in a general position and half a di­amide mol­ecule, the latter being located about a centre of inversion. In the acid, the carb­oxy­lic acid group is twisted out of the plane of the benzene ring to which it is attached [dihedral angle = 28.51 (8)°] and the carbonyl O atom and methyl group lie approximately to the same side of the mol­ecule [hy­droxy-O—C—C—C(H) torsion angle = −27.92 (17)°]. In the di­amide, the central C_4_N_2_O_2_ core is almost planar (r.m.s. deviation = 0.031 Å), and the pyridyl rings are perpendicular, lying to either side of the central plane [central residue/pyridyl dihedral angle = 88.60 (5)°]. In the mol­ecular packing, three-mol­ecule aggregates are formed *via* hy­droxy-O—H⋯N(pyrid­yl) hydrogen bonds. These are connected into a supra­molecular layer parallel to (12) *via* amide-N—H⋯O(carbon­yl) hydrogen bonds, as well as methyl­ene-C—H⋯O(amide) inter­actions. Significant π–π inter­actions occur between benzene/benzene, pyrid­yl/benzene and pyrid­yl/pyridyl rings within and between layers to consolidate the three-dimensional packing.

## Abstract 2

The 2:1 acid/di­amide co-crystal sees the components connected into three-mol­ecule aggregates *via* hy­droxy-O—H⋯N(pyrid­yl) hydrogen bonds. The aggregates are linked into a supra­molecular layer *via* amide-N—H⋯O(carbon­yl) and methyl­ene-C—H⋯O(amide) inter­actions. The three-dimensional packing is consolidated by π–π inter­actions involving all the aromatic residues.

## Chemical context   

Multi-component crystals, incorporating co-crystals, salts and co-crystal salts, attract continuing inter­est for a wide variety of applications as this technology may be employed, for example, to provide additives to promote the growth of crystals, to stabilize unusual and unstable coformers, to generate new luminescent materials, to separate enanti­omers, to facilitate absolute structure determination where the mol­ecule of concern does not have a significant anomalous scatterer, *etc.* (Aakeröy, 2015 ▸; Tiekink, 2012 ▸). Arguably, the areas attracting most inter­est in this context are the applications of multi-component crystals in the pharmaceutical industry (Duggirala *et al.*, 2016 ▸). Controlled/designed crystallization of multi-component crystals requires reliable synthon formation between the various components and that, of course, is the challenge of crystal engineering, let alone engineering small aggregates within crystals (Tiekink, 2014 ▸).

Systematic work on synthon propensities in multi-component crystals have revealed that carb­oxy­lic acids have a great likelihood of forming hy­droxy-O—H⋯N hydrogen bonds when co-crystallized with mol­ecules with pyridyl residues (Shattock *et al.*, 2008 ▸). A plausible explanation for this reliability is the formation of a supporting carbonyl-O⋯H inter­action involving the hydrogen atom adjacent to the pyridyl-nitro­gen atom. Indeed, in the absence of competing hydrogen-bonding functionality, the resulting seven-membered {⋯HOCO⋯HCN} heterosynthon is formed in more than 98% of relevant crystal structures (Shattock *et al.*, 2008 ▸). Recent systematic work in this phenomenon relates to mol­ecules shown in Scheme 1, where isomeric mol­ecules with two pyridyl rings separated by a di­amide residue have been co-crystallized with various carb­oxy­lic acids (Arman, Miller *et al.*, 2012 ▸; Arman *et al.*, 2013 ▸, Syed *et al.*, 2016 ▸; Jotani *et al.*, 2016 ▸). As a continuation of these studies, the title 2:1 co-crystal was isolated and characterized crystallographically and by Hirshfeld surface analysis.
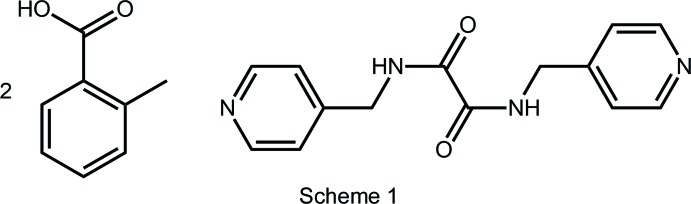


## Structural commentary   

**Figure 1:**
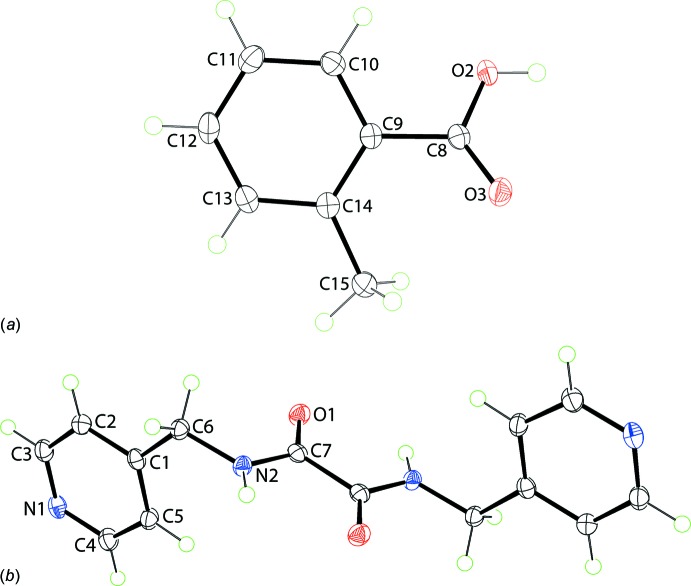
The mol­ecular structures of the mol­ecules comprising the title co-crystal showing the atom-labelling scheme and displacement ellipsoids at the 50% probability level: (*a*) 2-methyl­benzoic acid and (*b*) *N*,*N*′-bis­(pyridin-4-ylmeth­yl)ethanedi­amide; unlabelled atoms in the di­amide are generated by the symmetry operation (−1 − *x*, 2 − *y*, 1 − *z*).

The title co-crystal, Fig. 1 ▸, was formed from the 1:1 co-crystallization of 2-methyl­benzoic acid (hereafter, the acid) and *N*,*N*′-bis­(pyridin-4-ylmeth­yl)ethanedi­amide (hereafter, the di­amide) conducted in ethanol solution. The asymmetric unit comprises a full acid mol­ecule in a general position and half a di­amide mol­ecule, located about a centre of inversion, so the co-crystal is formulated as a 2:1 acid:di­amide co-crystal.

In the acid, the carb­oxy­lic acid group is twisted out of the plane of the benzene ring to which it is attached with the O3—C8—C9—C10 torsion angle being 150.23 (14)°, and, to a first approximation, with the carbonyl-O3 atom and methyl group lying to the same side of the mol­ecule as indicated in the O2—C8—C9—C10 torsion angle of −27.92 (17)°. The structure of the parent acid and several co-crystals featuring coformers shown in Scheme 2 are available for comparison; data are collected in Table 1 ▸. The common feature of all structures is the relative orientation of the carbonyl-O and methyl groups. Twists in the acid mol­ecules vary from almost co-planar to the situation found in the title co-crystal, with an even split of conformations amongst the six known co-crystal structures.
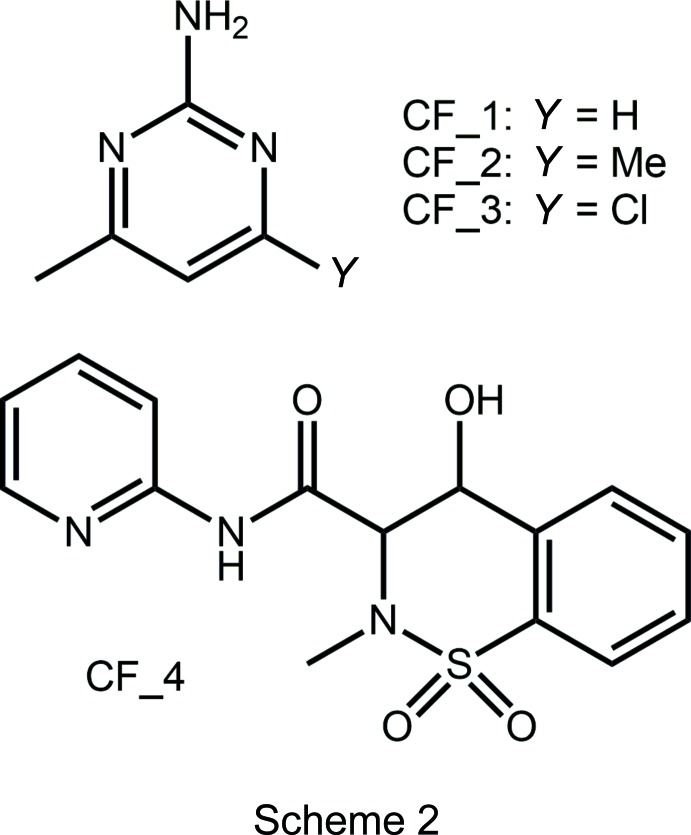


In the centrosymmetric di­amide, the central C_4_N_2_O_2_ core is essentially planar with an r.m.s. deviation (O1, N2, C6, C7 and symmetry equivalents) = 0.031 Å. This arrangement facilitates the formation of an intra­molecular amide-N—H⋯O(amide) hydrogen bond, Table 2 ▸. The pyridyl rings occupy positions on opposite sides of the central residue and project almost prime to this with the central residue/pyridyl dihedral angle being 88.60 (5)°. The aforementioned structural features match literature precedents, *i.e*. the two polymorphic forms of the parent di­amide and the di­amide in co-crystals with carb­oxy­lic acids and in a salt with a carboxyl­ate, Table 3 ▸. Finally, the central C—C bond length, considered long for a C*sp* ^2^—C*sp* ^2^ bond (Spek, 2009 ▸), matches the structural data included in Table 3 ▸; see Scheme 3 for chemical diagrams of coformers.
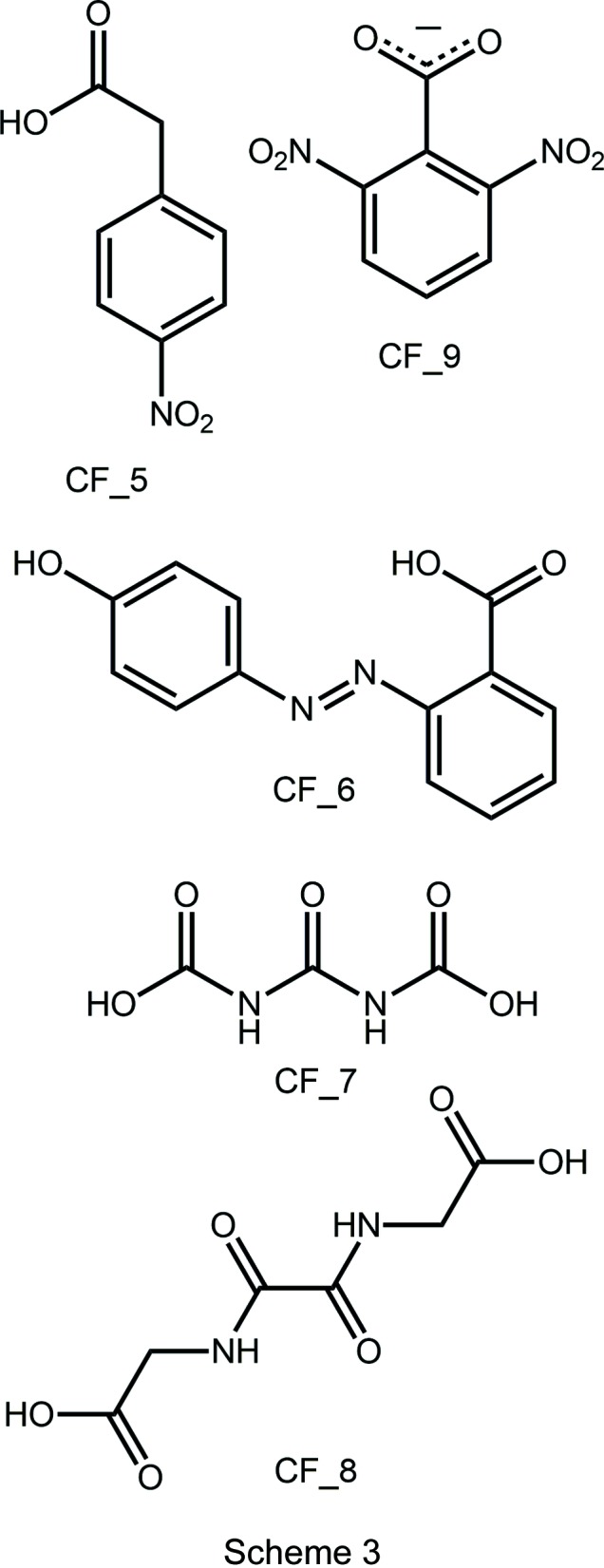


## Supra­molecular features   

**Figure 2:**
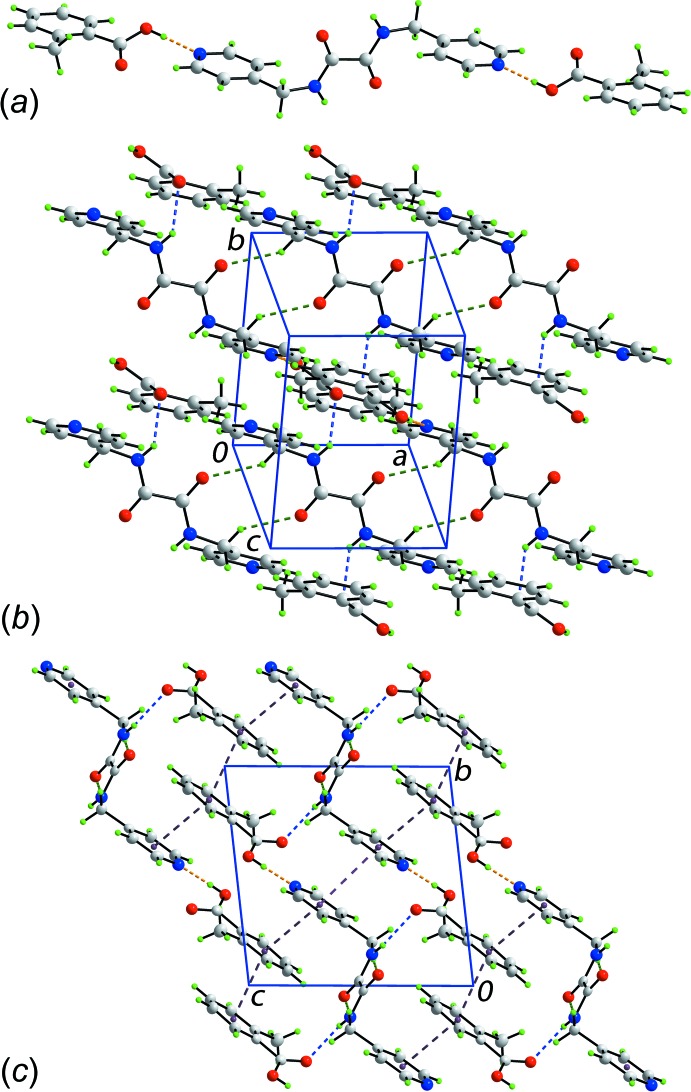
Mol­ecular packing in the title co-crystal: (*a*) three-mol­ecule aggregate sustained by hy­droxy-O—H⋯N(pyrid­yl) hydrogen bonds, (*b*) supra­molecular layers whereby the aggregates in (*a*) are connected by amide-N—H⋯O(carbon­yl) and methyl­ene-C—H⋯O(amide) inter­actions, and (*c*) a view of the unit-cell contents shown in projection down the *a* axis, highlighting the intra- and inter-layer π–π inter­actions to consolidate a three-dimensional architecture. The O—H⋯N, N—H⋯O, C—H⋯O and π–π inter­actions are shown as orange, blue, green and purple dashed lines, respectively.

The mol­ecular packing of the title co-crystal is dominated by hydrogen bonding, detailed in Table 2 ▸. The acid is connected to the di­amide *via* hy­droxy-O—H⋯N(pyrid­yl) hydrogen bonds to form a three-mol­ecule aggregate, Fig. 2 ▸ *a*. The inter­acting residues are not co-planar with the dihedral angle between the pyridyl and three CO_2_ groups being 25.67 (8)° so that the carbonyl-O3⋯H3 distance is 2.60 Å. This suggests only a minor role for the putative seven-membered heterosynthon {⋯OCOH⋯NCH} mentioned in the *Chemical context* and is consistent with the significant hydrogen-bonding inter­action involving the carbonyl-O3 atom to another residue. Indeed, the three-mol­ecule aggregates are connected into a supra­molecular layer parallel to (12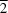) *via* amide-N—H⋯O(carbon­yl) hydrogen bonds as well as methyl­ene-C—H⋯O(amide) inter­actions, Fig. 2 ▸ *b*. Within layers, π–π inter­actions occur between pyridyl rings, and between layers additional π–π inter­actions occur between pyrid­yl/benzene and benzene/benzene rings to consolidate the three-dimensional packing, Table 4 ▸ and Fig. 2 ▸ *c*. Globally, the packing may be described as comprising alternating layers of aromatic rings and non-aromatic residues.

## Analysis of the Hirshfeld surfaces   

*Crystal Explorer 3.1* (Wolff *et al.*, 2012 ▸) was used to generate Hirshfeld surfaces mapped over *d*_norm_, *d*_e_, electrostatic potential, shape-index and curvedness for the title 2:1 co-crystal. The electrostatic potentials were calculated using *TONTO* (Spackman *et al.*, 2008 ▸; Jayatilaka *et al.*, 2005 ▸) integrated with *Crystal Explorer*, using the experimentally determined geometry as the input. Further, the electrostatic potentials were mapped on Hirshfeld surfaces using the STO-3G basis set at Hartree–Fock theory over a range ±0.15 au. The contact distances *d*_i_ and *d*_e_ from the Hirshfeld surface to the nearest atom inside and outside, respectively, enabled the analysis of the inter­molecular inter­actions through the mapping of *d*_norm_. The combination of *d*_i_ and *d*_e_ in the form of a two-dimensional fingerprint plot (Rohl *et al.*, 2008 ▸) provides a summary of the inter­molecular contacts.

**Figure 3:**
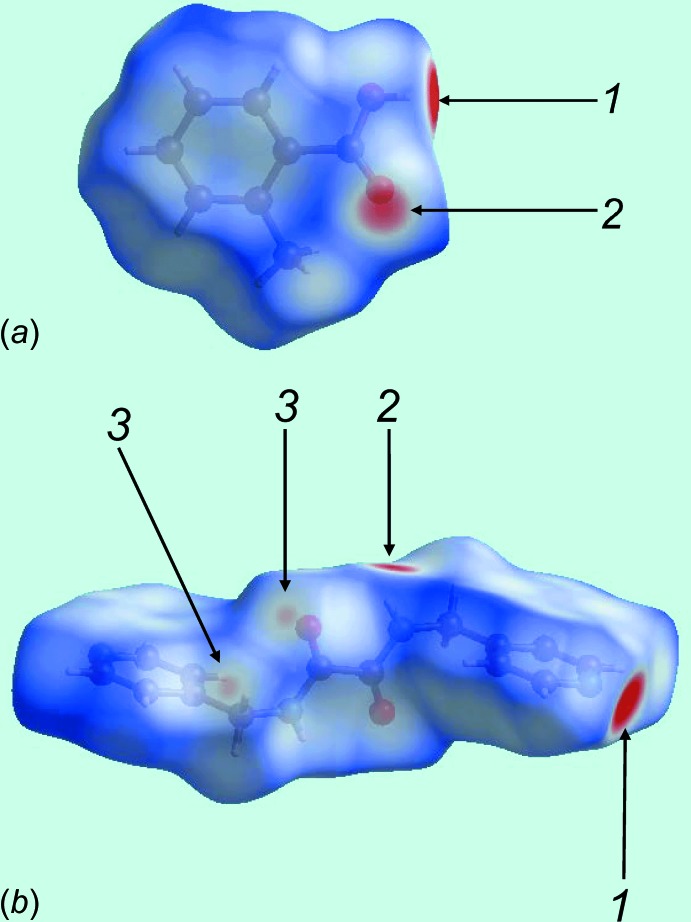
Views of the Hirshfeld surfaces mapped over *d*_norm_: (*a*) acid and (*b*) di­amide in the title 2:1 co-crystal. The contact points (red) are labelled to indicate the atoms participating in the inter­molecular inter­actions.

**Figure 4:**
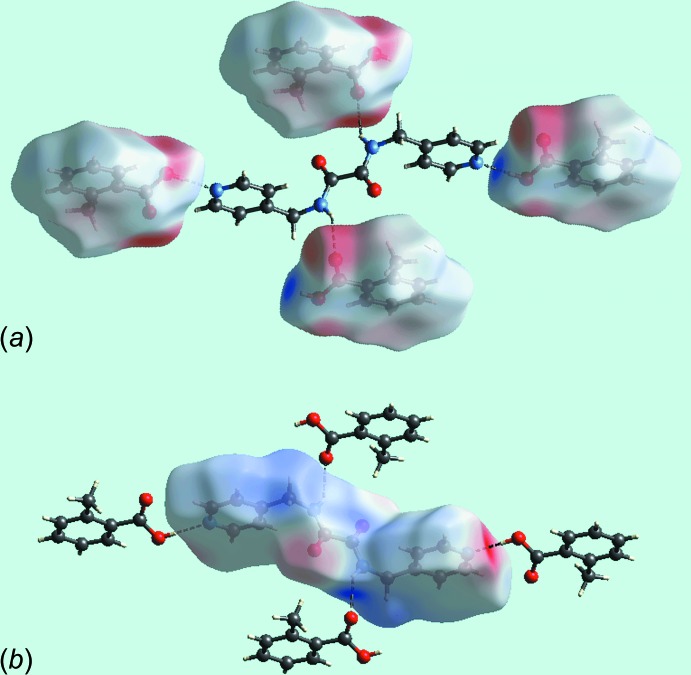
Hirshfeld surfaces mapped over *d*_norm_ showing hydrogen bonds with neighbouring mol­ecules with the reference mol­ecule being the (*a*) acid and (*b*) di­amide.

**Figure 5:**
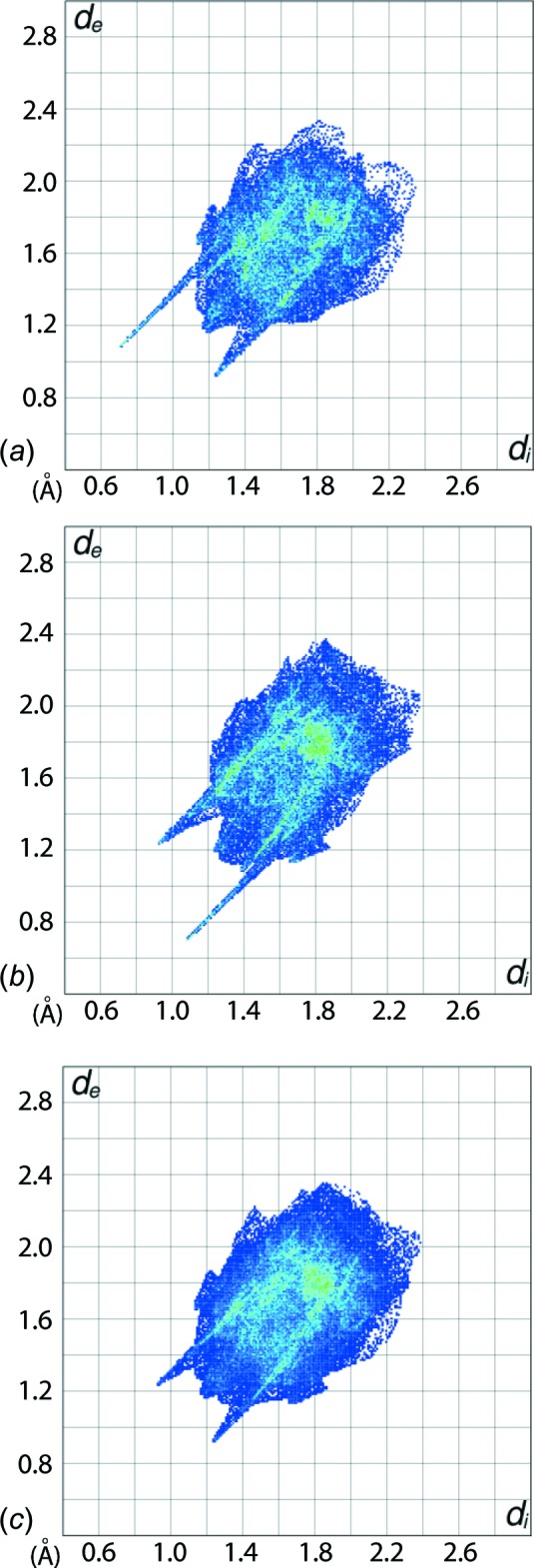
The two-dimensional fingerprint plots for the (*a*) acid, (*b*) di­amide, and (*c*) overall 2:1 co-crystal.

The strong hy­droxy-O—H⋯N(pyrid­yl) and amide-N—H⋯O(carbon­yl) inter­actions between the acid and di­amide mol­ecules are visualized as bright-red spots at the respective donor and acceptor atoms on the Hirshfeld surfaces mapped over *d*_norm_, and labelled as 1 and 2 in Fig. 3 ▸. The inter­molecular methyl­ene-C—H⋯O(amide) inter­actions appears as faint-red spots in Fig. 3 ▸ *b*, marked with a ‘3’. The immediate environment about each mol­ecule highlighting close contacts to the Hirshfeld surface by neighbouring mol­ecules is shown in Fig. 4 ▸. The full fingerprint (FP) plots showing various crystal packing inter­actions in the acid, di­amide and 2:1 co-crystal are shown in Fig. 5 ▸; the contributions from various contacts are listed in Table 5 ▸.

The prominent long spike at *d*_e_ + *d*_i_ ∼1.8 Å in the upper left (donor) region for the FP plot of the acid corresponds to H⋯N contacts and the spike at the same distance in the lower right (acceptor) region of the FP plot for the di­amide are the result of hy­droxy-O—H⋯N(pyrid­yl) inter­actions, Fig. 5 ▸ *a* and *b*, respectively. However, these spikes are not apparent in the overall FP for the 2:1 co-crystal as they no longer contribute to the surface of the resultant aggregate, Fig. 5 ▸ *c*. Pairs of somewhat blunted spikes corresponding to N⋯H/H⋯N contacts at *d*_e_ + *d*_i_ ∼ 2.9 Å result from amide-N—H⋯O(carbon­yl) inter­actions between the acid and di­amide mol­ecules are evident in the overall FP, Fig. 5 ▸ *c*.

The O⋯H/H⋯O contacts, which make a significant contribution to the mol­ecular packing, show different characteristic features in the respective delineated FP plots of the acid and di­amide. For the acid, Fig. 5 ▸ *a*, a long prominent spike at *d*_e_ + *d*_i_ ∼ 2.5 Å in the acceptor region corresponds to a 6.6% contribution from H⋯O contacts to the Hirshfeld surface, and a short spike at *d*_e_ + *d*_i_ ∼ 2.15 Å in the donor region with a 14.0% contribution. The reverse situation is observed for the di­amide mol­ecule wherein the FP plot, Fig. 5 ▸ *b*, contains a long prominent spike in the donor region and the short spike in the acceptor at the same *d*_e_ + *d*_i_ distance, and with 10.7 and 14.9% contributions from O⋯H and H⋯O contacts, respectively.

**Figure 6:**
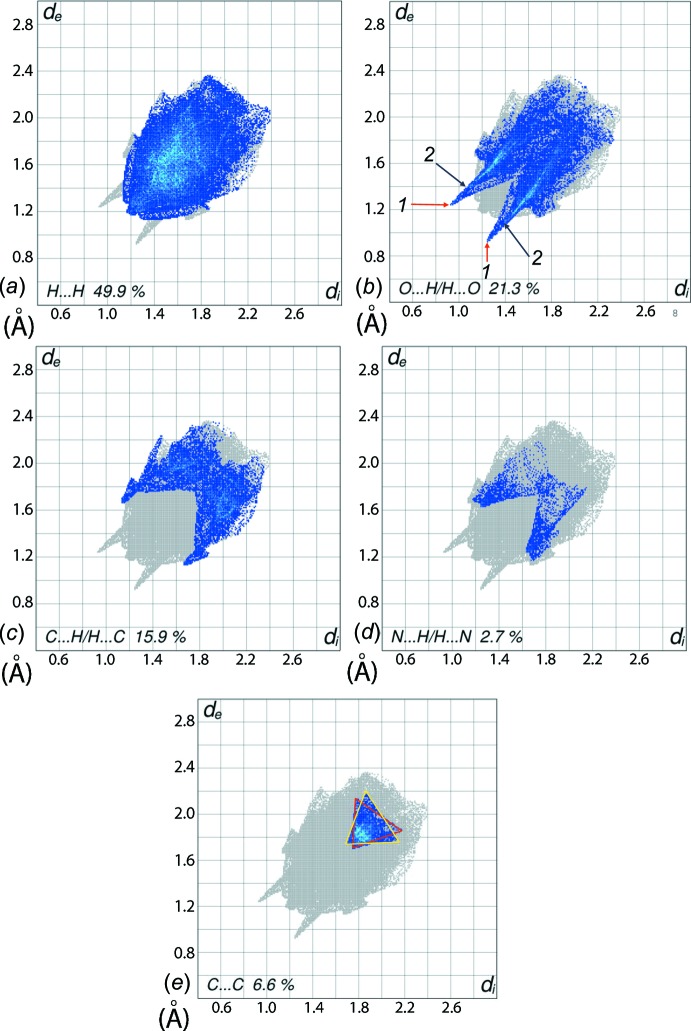
The two-dimensional fingerprint plot for the title 2:1 co-crystal showing contributions from different contacts: (*a*) H⋯H, (*b*) O⋯H/H⋯O, (*c*) C⋯H/H⋯C, (*d*) N⋯H/H⋯N, and (*e*) C⋯C.

FP plots for the co-crystal delineated into H⋯H, O⋯H/H⋯O, C⋯H/H⋯C, N⋯H/H⋯N and C⋯C are shown in Fig. 6 ▸ *a*–*e*, respectively. The H⋯H contacts appear as asymmetrically scattered points covering a large region of the FP plot with a single broad peak at *d*_e_ = *d*_i_ ∼ 1.2 Å for each of the co-crystal constituents, with percentage contributions of 48.7 and 45.7% for the acid and di­amide mol­ecules, respectively. The overall 49.9% contribution to Hirshfeld surface of the co-crystal results in nearly symmetric through the superimposition of individual fingerprint plots, Fig. 6 ▸ *a*.

**Figure 7:**
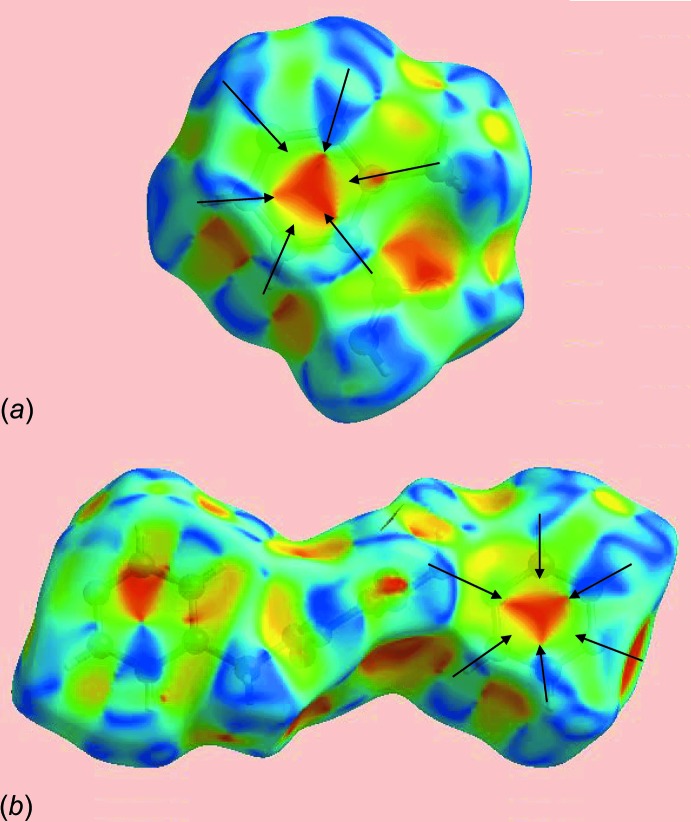
Hirshfeld surfaces mapped over the shape index for (*a*) the acid and (*b*) the di­amide, highlighting the regions involved in π–π stacking inter­actions.

**Figure 8:**
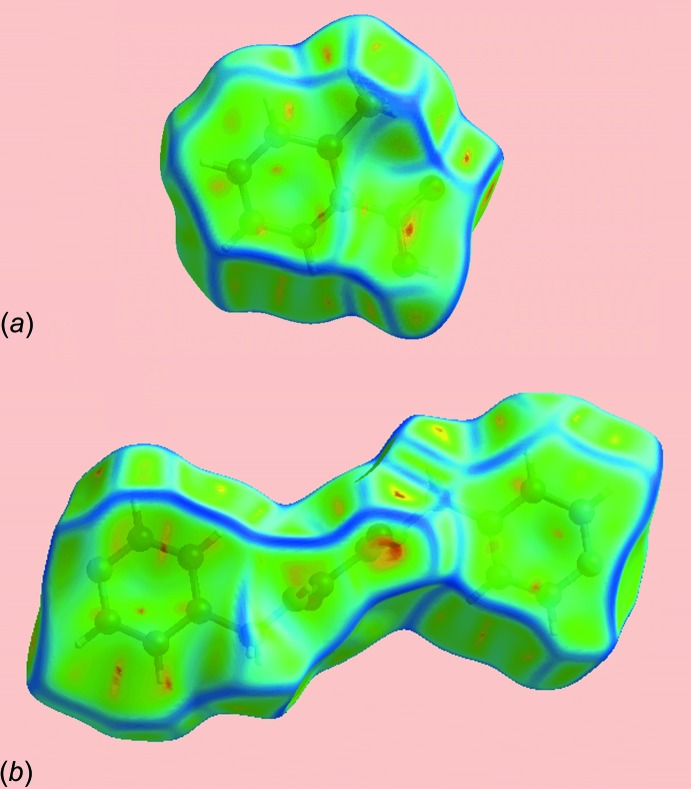
Hirshfeld surfaces mapped over curvedness for (*a*) the acid and (*b*) the di­amide, highlighting the regions involved in π–π stacking inter­actions.

The FP plot for O⋯H/H⋯O contacts, Fig. 6 ▸ *b*, has two pairs of spikes superimposed in the (*d*_e_, *d*_i_) region with minimum *d*_e_ + *d*_i_ distances ∼ 2.2 and 2.5 Å. These correspond to a 21.3% contribution to the Hirshfeld surface, and reflect the presence of inter­molecular N—H⋯O and C—H⋯O inter­actions, identified with labels 1 and 2 in Fig. 6 ▸ *b*. The 15.9% contribution from the C⋯H/H⋯C contacts to the Hirshfeld surface results in a symmetric pair of wings, Fig. 6 ▸ *c*. The FP plot corresponding to C⋯C contacts, Fig. 6 ▸ *e*, in the (*d*_e_, *d*_i_) region between 1.7 to 2.2 Å appears as the two distinct, overlapping triangles identified with red and yellow boundaries in Fig. 6 ▸ *e*, and shows two types of π–π stacking inter­actions: one between dissimilar rings (pyridyl and benzene) and the other between symmetry-related rings (benzene and benzene, and pyridyl and pyrid­yl). The presence of these π–π stacking inter­actions is also indicated by the appearance of red and blue triangles on the shape-indexed surfaces identified with arrows in the images of Fig. 7 ▸, and in the flat regions on the Hirshfeld surfaces mapped with curvedness in Fig. 8 ▸.

The inter­molecular inter­actions were further assessed by using the enrichment ratio, ER (Jelsch *et al.*, 2014 ▸). This is a relatively new descriptor and is based on Hirshfeld surface analysis. The ER for the co-crystal together with those for the acid and di­amide mol­ecules are listed in Table 6 ▸. The largest contribution to the Hirshfeld surfaces are from H⋯H contacts, Table 5 ▸, and their respective ER values are close to unity. This shows that the contribution from dispersive forces are significant in the mol­ecule packing of the title 2:1 co-crystal, in contrast to that observed in a related, recently published structure, namely, the salt [2-({[(pyridin-1-ium-2-yl­meth­yl)carbamo­yl]formamido}­meth­yl)-pyridin-1-ium][3,5-di­carb­oxy­benzoate], *i.e.* containing the diprotonated form of the isomeric 2-pyridyl-containing di­amide (Syed *et al.*, 2016 ▸). In the latter, O⋯H/H⋯O contacts make the greatest contribution to the crystal packing. It is the presence of different substituents in the benzene ring in the acid mol­ecule in the co-crystal, *i.e*. methyl, as opposed to carb­oxy­lic acid/carboxyl­ate groups in the salt, that provides an explanation for this difference. The ER value for O⋯H/H⋯O contacts, *i.e*. 1.30, shows the propensity to form hy­droxy-O—H⋯N(pyrid­yl) and amide-N—H⋯O(carbon­yl) hydrogen bonds as well as methyl­ene-C—H⋯O(amide) inter­actions. The formation of extensive π–π inter­actions is reflected in the relatively high ER values corresponding/related to C⋯C contacts, Table 6 ▸. The absence of C—H⋯π and related inter­actions is reflected in low ER values, *i.e*. < 0.8. Conversely, the N⋯H/H⋯N contacts in a crystal having ER values equal to greater than or equal to unity for the acid/di­amide mol­ecules reduces to 0.84 in the 2:1 co-crystal, indicating a reduced likelihood of formation once the co-crystal is stabil­ized by other inter­actions. The enrichment ratios for other contacts are of low significance as they are derived from less important inter­actions which have small contributions to Hirshfeld surfaces.

## Database survey   

As mentioned in the *Chemical context*, the di­amide in the title 2:1 co-crystal and isomeric forms have attracted considerable inter­est in the crystal engineering community no doubt owing to the variable functional groups and conformational flexibility. Indeed, the di­amide in the title co-crystal featured in early studies of halogen I⋯N halogen bonding (Goroff *et al.*, 2005 ▸). Over and above these investigations, the role of the di­amide in coordination chemistry has also been studied. Bidentate bridging is the prominent coordination mode observed in both neutral, *e.g*. [HgI_2_(di­amide)]_*n*_ (Zeng *et al.*, 2008 ▸) and charged, *e.g*. polymeric [Ag(di­amide)NO_3_]_*n*_ (Schauer *et al.*, 1998 ▸) and oligiomeric {[Ph_2_PCH_2_PPh_2_Au_2_(di­amide)]_2_(ClO_4_)_4_(EtOEt)_4_} (Tzeng *et al.*, 2006 ▸), species.

## Synthesis and crystallization   

The di­amide (0.2 g), prepared in accord with the literature procedure (Schauer *et al.*, 1997 ▸), in ethanol (10 ml) was added to a ethanol solution (10 ml) of 2-methyl­benzoic acid (Merck, 0.1 g). The mixture was stirred for 1 h at room temperature after which a white precipitate was deposited. The solution was filtered by vacuum suction, and the filtrate was then left to stand under ambient conditions, yielding colourless prisms after 2 weeks.

## Refinement   

Crystal data, data collection and structure refinement details are summarized in Table 7 ▸. The carbon-bound H-atoms were placed in calculated positions (C—H = 0.95–0.99 Å) and were included in the refinement in the riding-model approximation, with *U*_iso_(H) set to 1.2*U*_eq_(C). The oxygen- and nitro­gen-bound H-atoms were located in a difference Fourier map but were refined with distance restraints of O—H = 0.84±0.01 Å and N—H = 0.88±0.01 Å, and with *U*_iso_(H) set to 1.5*U*_eq_(O) and 1.2*U*_eq_(N).

## Supplementary Material

Crystal structure: contains datablock(s) I, global. DOI: 10.1107/S2056989016002735/hb7566sup1.cif

Structure factors: contains datablock(s) I. DOI: 10.1107/S2056989016002735/hb7566Isup2.hkl

Supporting information file. DOI: 10.1107/S2056989016002735/hb7566Isup3.cml

CCDC reference: 1453604

Additional supporting information:  crystallographic information; 3D view; checkCIF report

## Figures and Tables

**Table 1:** Dihedral and torsion angles (°) for 2-methyl­benzoic acid in the title co-crystal and in literature precedents

Compound	C_H_—C—C—OH	C_6_/CO_2_	CSD Refcode*^*b*^*	Reference
Parent compound	1.7 (2)	1.5 (5)	OTOLIC02	Thakur & Desiraju (2008 ▸)
1:1 Co-crystal with CF_1	7.5 (2)	8.04 (9)	WICZUF	Day *et al.* (2009 ▸)
1:1 Co-crystal with CF_2	4.25 (19)	4.02 (12)	EXIBOD	Ebenezer *et al.* (2011 ▸)
1:1 Co-crystal with CF_3	27.4 (3)	27.8 (2)	EXIZIR	Ebenezer *et al.* (2011 ▸)
1:1 Co-crystal with CF_4	23.0 (2)	23.86 (8)	CEKLEL	Wales *et al.* (2012 ▸)
Title co-crystal	−27.92 (18)	28.51 (8)	–	This work

Notes: (*a*) Refer to Scheme 2 for the chemical structures of coformers CF_1–CF_4. (*b*) Groom & Allen (2014 ▸).

**Table 2:** Hydrogen-bond geometry (Å, °)

*D*—H⋯*A*	*D*—H	H⋯*A*	*D*⋯*A*	*D*—H⋯*A*
N2—H2*N*⋯O1^i^	0.87 (1)	2.31 (1)	2.7100 (16)	108 (1)
O2—H2*O*⋯N1	0.85 (2)	1.79 (2)	2.6378 (16)	178 (2)
N2—H2*N*⋯O3^ii^	0.87 (1)	2.17 (1)	2.8933 (15)	140 (1)
C6—H3*B*⋯O1^iii^	0.99	2.48	3.3461 (18)	146

Symmetry codes: (i) 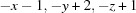; (ii) 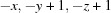; (iii) 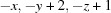.

**Table 3:** Selected geometric details (Å, °) for *N*,*N*′-bis­(pyridin-4-ylmeth­yl)ethanedi­amide mol­ecules and protonated forms*^*a*^*

Coformer	C_4_N_2_O_2_/N-ring	C(=O)—C(=O)	Refcode*^*b*^*	Ref.
–^*c*,*d*^	74.90 (4)	1.532 (2)	CICYOD01	Lee (2010 ▸)
–*^*e*^*	68.83 (4); 70.89 (5)	1.541 (3)	CICYOD	Lee & Wang (2007 ▸)
	80.46 (5); 83.35 (6)	1.541 (3)		
CF_5^*c*,*f*^	87.37 (4)	1.534 (2)	NAXMEG	Arman, Kaulgud *et al.* (2012 ▸)
CF_6^*c*,*f*^	79.86 (4)	1.542 (2)	AJEZEV	Arman *et al.* (2009 ▸)
CF_7^*g*^	70.50 (4); 76.89 (4)	1.52 (2)	CAJRAH	Nguyen *et al.* (2001 ▸)
CF_8^*c*,*g*,*h*^	73.38 (11)	1.523 (7)	SEPSIP	Nguyen *et al.* (1998 ▸)
CF_8^*c*,*g*,*i*^	72.87 (9)	1.514 (5)	SEPSIP01	Nguyen *et al.* (2001 ▸)
CF_9^*c*,*f*^	75.83 (5)	1.543 (3)	TIPGUW	Arman *et al.* (2013 ▸)
2-Methyl­benzoic acid	88.66 (4)	1.5356 (19)	–	This work

Notes: (*a*) Refer to Scheme 3 for the chemical structures of coformers, CF_5–CF_9; (*b*) Groom & Allen (2014 ▸); (*c*) mol­ecule/dianion is centrosymmetric; (*d*) form I; (*e*) form II (two independent mol­ecules); (*f*) 2:1 carb­oxy­lic acid/carboxyl­ate di­amide co-crystal/salt; (*g*) 1:1 di­carb­oxy­lic acid di­amide co-crystal; (*h*) form I; (*i*) form II.

**Table 4:** π–π Inter­actions (Å, °)

Ring 1	Ring 2	Inter-centroid distance	Dihedral angle	Symmetry
N1,C1–C5	N1,C1–C5	3.5980 (8)	0	−*x*, 1 − *y*, 1 − *z*
N1,C1–C5	C9–C14	3.7833 (9)	4.63 (7)	1 − *x*, 1 − *y*, −*z*
C9–C14	C9–C14	3.8473 (8)	0	−1 − *x*, −*y*, −*z*

**Table 5:** Major percentage contribution of the different inter­molecular inter­actions to the Hirshfeld surfaces for the acid, di­amide and 2:1 co-crystal

Contact	Acid	Di­amide	Co-crystal
H⋯H	48.7	45.2	49.9
O⋯H/H⋯O	20.6	25.6	21.3
C⋯H/H⋯C	16.7	12.0	15.9
N⋯H/H⋯N	3.8	8.9	2.7
C⋯C	5.9	6.4	6.6

**Table 6:** Enrichment ratios (ER) for the acid, di­amide and co-crystal

Inter­action	Acid	Di­amide	Co-crystal
H⋯H	1.02	0.97	1.02
O⋯H/H⋯O	1.22	1.46	1.30
C⋯C	2.30	3.60	2.55
C⋯H/H⋯C	0.75	0.66	0.71
N⋯H/H⋯N	1.06	1.20	0.84

**Table 7:** Experimental details

Crystal data	
Chemical formula	C_14_H_14_N_4_O_2_·2C_8_H_8_O_2_
*M*_r_	542.58
Crystal system, space group	Triclinic, *P* 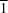
Temperature (K)	100
*a*, *b*, *c* (Å)	6.8948 (5), 9.7219 (5), 9.9621 (7)
α, β, γ (°)	82.971 (5), 81.638 (6), 85.686 (5)
*V* (Å^3^)	654.58 (8)
*Z*	1
Radiation type	Mo *K*α
μ (mm^−1^)	0.10
Crystal size (mm)	0.21 × 0.15 × 0.10

Data collection	
Diffractometer	Agilent Technologies SuperNova Dual diffractometer with an Atlas detector
Absorption correction	Multi-scan (*CrysAlis PRO*; Agilent, 2014 ▸)
*T*_min_, *T*_max_	0.580, 1.000
No. of measured, independent and observed [*I* > 2σ(*I*)] reflections	15067, 2993, 2358
*R*_int_	0.044
(sin θ/λ)_max_ (Å^−1^)	0.650

Refinement	
*R*[*F* ^2^ > 2σ(*F* ^2^)], *wR*(*F* ^2^), *S*	0.041, 0.106, 1.06
No. of reflections	2993
No. of parameters	188
No. of restraints	2
Δρ_max_, Δρ_min_ (e Å^−3^)	0.34, −0.23

Computer programs: *CrysAlis PRO* (Agilent, 2014 ▸), *SHELXS97* (Sheldrick, 2008 ▸), *SHELXL2014* (Sheldrick, 2015 ▸), *ORTEP-3 for Windows* (Farrugia, 2012 ▸), *DIAMOND* (Brandenburg, 2006 ▸) and *publCIF* (Westrip, 2010 ▸).

## supplementary crystallographic information

### Crystal data

**Table:**

C_14_H_14_N_4_O_2_·2C_8_H_8_O_2_	*Z* = 1
*M**_r_* = 542.58	*F*(000) = 286
Triclinic, *P*1	*D*_x_ = 1.376 Mg m^−^^3^
*a* = 6.8948 (5) Å	Mo *K*α radiation, λ = 0.71073 Å
*b* = 9.7219 (5) Å	Cell parameters from 3840 reflections
*c* = 9.9621 (7) Å	θ = 3.5–30.0°
α = 82.971 (5)°	µ = 0.10 mm^−^^1^
β = 81.638 (6)°	*T* = 100 K
γ = 85.686 (5)°	Prism, colourless
*V* = 654.58 (8) Å^3^	0.21 × 0.15 × 0.10 mm

### Data collection

**Table:**

Agilent Technologies SuperNova Dual diffractometer with an Atlas detector	2993 independent reflections
Radiation source: SuperNova (Mo) X-ray Source	2358 reflections with *I* > 2σ(*I*)
Mirror monochromator	*R*_int_ = 0.044
Detector resolution: 10.4041 pixels mm^-1^	θ_max_ = 27.5°, θ_min_ = 3.0°
ω scan	*h* = −8→8
Absorption correction: multi-scan (*CrysAlis PRO*; Agilent, 2014)	*k* = −12→12
*T*_min_ = 0.580, *T*_max_ = 1.000	*l* = −12→12
15067 measured reflections	

### Refinement

**Table:**

Refinement on *F*^2^	2 restraints
Least-squares matrix: full	Hydrogen site location: mixed
*R*[*F*^2^ > 2σ(*F*^2^)] = 0.041	*w* = 1/[σ^2^(*F*_o_^2^) + (0.0434*P*)^2^ + 0.2225*P*] where *P* = (*F*_o_^2^ + 2*F*_c_^2^)/3
*wR*(*F*^2^) = 0.106	(Δ/σ)_max_ < 0.001
*S* = 1.06	Δρ_max_ = 0.34 e Å^−^^3^
2993 reflections	Δρ_min_ = −0.23 e Å^−^^3^
188 parameters	

### Special details

**Table:**

Geometry. All esds (except the esd in the dihedral angle between two l.s. planes) are estimated using the full covariance matrix. The cell esds are taken into account individually in the estimation of esds in distances, angles and torsion angles; correlations between esds in cell parameters are only used when they are defined by crystal symmetry. An approximate (isotropic) treatment of cell esds is used for estimating esds involving l.s. planes.

### Fractional atomic coordinates and isotropic or equivalent isotropic displacement parameters (Å^2^)

**Table:**

	*x*	*y*	*z*	*U*_iso_*/*U*_eq_	
O1	−0.26845 (15)	1.04906 (10)	0.41903 (10)	0.0202 (2)	
N1	0.10595 (19)	0.54943 (12)	0.25502 (12)	0.0194 (3)	
N2	−0.34990 (18)	0.85635 (11)	0.56442 (12)	0.0157 (3)	
H2N	−0.4483 (18)	0.8165 (15)	0.6161 (14)	0.019*	
C1	−0.0650 (2)	0.71369 (13)	0.45691 (14)	0.0159 (3)	
C2	0.1324 (2)	0.66915 (14)	0.44690 (15)	0.0183 (3)	
H2	0.2123	0.6945	0.5089	0.022*	
C3	0.2113 (2)	0.58766 (14)	0.34574 (15)	0.0197 (3)	
H3	0.3461	0.5574	0.3403	0.024*	
C4	−0.0837 (2)	0.59250 (14)	0.26467 (15)	0.0205 (3)	
H4	−0.1600	0.5661	0.2009	0.025*	
C5	−0.1746 (2)	0.67411 (14)	0.36357 (15)	0.0182 (3)	
H5	−0.3100	0.7024	0.3672	0.022*	
C6	−0.1494 (2)	0.80107 (14)	0.57005 (14)	0.0165 (3)	
H3A	−0.1442	0.7438	0.6589	0.020*	
H3B	−0.0648	0.8796	0.5668	0.020*	
C7	−0.3901 (2)	0.97768 (13)	0.49211 (14)	0.0154 (3)	
O2	0.30435 (15)	0.41238 (10)	0.06130 (11)	0.0200 (2)	
H2O	0.242 (2)	0.4581 (16)	0.1233 (15)	0.030*	
O3	0.50558 (15)	0.34690 (10)	0.21737 (10)	0.0210 (2)	
C8	0.4571 (2)	0.33987 (13)	0.10547 (14)	0.0160 (3)	
C9	0.5600 (2)	0.24530 (13)	0.00615 (14)	0.0157 (3)	
C10	0.4507 (2)	0.19550 (14)	−0.08313 (15)	0.0183 (3)	
H10	0.3160	0.2250	−0.0817	0.022*	
C11	0.5355 (2)	0.10374 (14)	−0.17385 (15)	0.0222 (3)	
H11	0.4586	0.0672	−0.2314	0.027*	
C12	0.7337 (2)	0.06622 (14)	−0.17932 (15)	0.0220 (3)	
H12	0.7947	0.0059	−0.2432	0.026*	
C13	0.8436 (2)	0.11621 (14)	−0.09211 (15)	0.0195 (3)	
H13	0.9800	0.0904	−0.0983	0.023*	
C14	0.7595 (2)	0.20349 (13)	0.00468 (14)	0.0166 (3)	
C15	0.8858 (2)	0.24942 (15)	0.09983 (16)	0.0225 (3)	
H15A	0.8762	0.3510	0.0945	0.034*	
H15B	1.0226	0.2173	0.0735	0.034*	
H15C	0.8409	0.2100	0.1935	0.034*	

### Atomic displacement parameters (Å^2^)

**Table:**

	*U*^11^	*U*^22^	*U*^33^	*U*^12^	*U*^13^	*U*^23^
O1	0.0158 (5)	0.0209 (5)	0.0229 (6)	−0.0017 (4)	−0.0010 (4)	−0.0004 (4)
N1	0.0224 (7)	0.0166 (6)	0.0179 (6)	0.0030 (5)	0.0001 (5)	−0.0027 (5)
N2	0.0132 (6)	0.0170 (6)	0.0166 (6)	0.0005 (4)	−0.0005 (5)	−0.0031 (4)
C1	0.0179 (7)	0.0129 (6)	0.0155 (7)	−0.0004 (5)	0.0001 (6)	0.0004 (5)
C2	0.0170 (7)	0.0173 (6)	0.0208 (7)	−0.0004 (5)	−0.0025 (6)	−0.0029 (5)
C3	0.0170 (7)	0.0164 (6)	0.0243 (8)	0.0011 (5)	0.0006 (6)	−0.0018 (6)
C4	0.0238 (8)	0.0190 (7)	0.0190 (7)	0.0025 (6)	−0.0048 (6)	−0.0037 (6)
C5	0.0160 (7)	0.0185 (7)	0.0199 (7)	0.0031 (5)	−0.0028 (6)	−0.0039 (5)
C6	0.0150 (7)	0.0176 (6)	0.0170 (7)	0.0011 (5)	−0.0021 (6)	−0.0035 (5)
C7	0.0176 (8)	0.0158 (6)	0.0137 (7)	−0.0006 (5)	−0.0019 (6)	−0.0058 (5)
O2	0.0177 (6)	0.0225 (5)	0.0196 (5)	0.0044 (4)	−0.0008 (4)	−0.0067 (4)
O3	0.0239 (6)	0.0232 (5)	0.0156 (5)	0.0019 (4)	−0.0012 (4)	−0.0042 (4)
C8	0.0154 (7)	0.0151 (6)	0.0163 (7)	−0.0020 (5)	0.0015 (6)	−0.0005 (5)
C9	0.0174 (7)	0.0144 (6)	0.0140 (7)	−0.0017 (5)	0.0009 (6)	0.0001 (5)
C10	0.0166 (7)	0.0197 (7)	0.0180 (7)	0.0005 (5)	−0.0023 (6)	−0.0008 (5)
C11	0.0283 (9)	0.0211 (7)	0.0185 (8)	−0.0015 (6)	−0.0062 (6)	−0.0039 (6)
C12	0.0296 (9)	0.0182 (7)	0.0172 (7)	0.0046 (6)	−0.0005 (6)	−0.0045 (6)
C13	0.0198 (8)	0.0186 (7)	0.0183 (7)	0.0028 (6)	0.0004 (6)	−0.0008 (5)
C14	0.0185 (7)	0.0140 (6)	0.0163 (7)	−0.0021 (5)	0.0000 (6)	0.0001 (5)
C15	0.0173 (8)	0.0259 (7)	0.0251 (8)	−0.0003 (6)	−0.0024 (6)	−0.0075 (6)

### Geometric parameters (Å, º)

**Table:**

O1—C7	1.2252 (17)	O2—C8	1.3217 (17)
N1—C4	1.3364 (19)	O2—H2O	0.853 (9)
N1—C3	1.3401 (19)	O3—C8	1.2205 (17)
N2—C7	1.3371 (17)	C8—C9	1.4994 (18)
N2—C6	1.4510 (18)	C9—C10	1.396 (2)
N2—H2N	0.874 (9)	C9—C14	1.403 (2)
C1—C5	1.385 (2)	C10—C11	1.385 (2)
C1—C2	1.390 (2)	C10—H10	0.9500
C1—C6	1.5166 (18)	C11—C12	1.383 (2)
C2—C3	1.3820 (19)	C11—H11	0.9500
C2—H2	0.9500	C12—C13	1.384 (2)
C3—H3	0.9500	C12—H12	0.9500
C4—C5	1.3892 (19)	C13—C14	1.3964 (19)
C4—H4	0.9500	C13—H13	0.9500
C5—H5	0.9500	C14—C15	1.503 (2)
C6—H3A	0.9900	C15—H15A	0.9800
C6—H3B	0.9900	C15—H15B	0.9800
C7—C7^i^	1.536 (3)	C15—H15C	0.9800
			
C4—N1—C3	117.67 (12)	C8—O2—H2O	110.8 (13)
C7—N2—C6	121.54 (12)	O3—C8—O2	123.13 (12)
C7—N2—H2N	117.2 (11)	O3—C8—C9	123.68 (13)
C6—N2—H2N	120.9 (11)	O2—C8—C9	113.16 (12)
C5—C1—C2	117.97 (13)	C10—C9—C14	120.20 (12)
C5—C1—C6	123.56 (13)	C10—C9—C8	118.28 (13)
C2—C1—C6	118.47 (13)	C14—C9—C8	121.48 (12)
C3—C2—C1	119.31 (14)	C11—C10—C9	121.07 (14)
C3—C2—H2	120.3	C11—C10—H10	119.5
C1—C2—H2	120.3	C9—C10—H10	119.5
N1—C3—C2	122.93 (14)	C12—C11—C10	118.97 (14)
N1—C3—H3	118.5	C12—C11—H11	120.5
C2—C3—H3	118.5	C10—C11—H11	120.5
N1—C4—C5	123.05 (14)	C11—C12—C13	120.28 (13)
N1—C4—H4	118.5	C11—C12—H12	119.9
C5—C4—H4	118.5	C13—C12—H12	119.9
C1—C5—C4	119.07 (13)	C12—C13—C14	121.79 (14)
C1—C5—H5	120.5	C12—C13—H13	119.1
C4—C5—H5	120.5	C14—C13—H13	119.1
N2—C6—C1	115.06 (12)	C13—C14—C9	117.56 (13)
N2—C6—H3A	108.5	C13—C14—C15	119.00 (13)
C1—C6—H3A	108.5	C9—C14—C15	123.43 (12)
N2—C6—H3B	108.5	C14—C15—H15A	109.5
C1—C6—H3B	108.5	C14—C15—H15B	109.5
H3A—C6—H3B	107.5	H15A—C15—H15B	109.5
O1—C7—N2	125.26 (13)	C14—C15—H15C	109.5
O1—C7—C7^i^	121.45 (15)	H15A—C15—H15C	109.5
N2—C7—C7^i^	113.29 (15)	H15B—C15—H15C	109.5
			
C5—C1—C2—C3	−0.2 (2)	O2—C8—C9—C10	−27.92 (17)
C6—C1—C2—C3	179.02 (12)	O3—C8—C9—C14	−27.8 (2)
C4—N1—C3—C2	−0.3 (2)	O2—C8—C9—C14	154.08 (12)
C1—C2—C3—N1	0.4 (2)	C14—C9—C10—C11	0.4 (2)
C3—N1—C4—C5	0.0 (2)	C8—C9—C10—C11	−177.60 (12)
C2—C1—C5—C4	−0.1 (2)	C9—C10—C11—C12	−2.8 (2)
C6—C1—C5—C4	−179.24 (12)	C10—C11—C12—C13	2.1 (2)
N1—C4—C5—C1	0.2 (2)	C11—C12—C13—C14	0.9 (2)
C7—N2—C6—C1	−87.65 (15)	C12—C13—C14—C9	−3.3 (2)
C5—C1—C6—N2	−7.47 (19)	C12—C13—C14—C15	177.62 (13)
C2—C1—C6—N2	173.38 (12)	C10—C9—C14—C13	2.56 (19)
C6—N2—C7—O1	4.4 (2)	C8—C9—C14—C13	−179.48 (12)
C6—N2—C7—C7^i^	−175.86 (13)	C10—C9—C14—C15	−178.36 (13)
O3—C8—C9—C10	150.23 (14)	C8—C9—C14—C15	−0.4 (2)

Symmetry code: (i) −*x*−1, −*y*+2, −*z*+1.

### Hydrogen-bond geometry (Å, º)

**Table:**

*D*—H···*A*	*D*—H	H···*A*	*D*···*A*	*D*—H···*A*
N2—H2*N*···O1^i^	0.87 (1)	2.31 (1)	2.7100 (16)	108 (1)
O2—H2*O*···N1	0.85 (2)	1.79 (2)	2.6378 (16)	178 (2)
N2—H2*N*···O3^ii^	0.87 (1)	2.17 (1)	2.8933 (15)	140 (1)
C6—H3*B*···O1^iii^	0.99	2.48	3.3461 (18)	146

Symmetry codes: (i) −*x*−1, −*y*+2, −*z*+1; (ii) −*x*, −*y*+1, −*z*+1; (iii) −*x*, −*y*+2, −*z*+1.
